# Quantitative Monitoring Maximizes Cost-Saving Strategies When Antagonizing Neuromuscular Block With Sugammadex

**DOI:** 10.7759/cureus.68551

**Published:** 2024-09-03

**Authors:** Steve Haberkorn, Mark Twite, Katherine Klockau, Gina Whitney, Debra J Faulk

**Affiliations:** 1 Department of Anesthesiology, University of Colorado, Aurora, USA; 2 Department of Pharmacy, Children's Hospital Colorado, Aurora, USA

**Keywords:** pediatric anesthesiology, sugammadex, antagonism, quantitative monitoring, neuromuscular block

## Abstract

Introduction

Weight-based dosing combined with variable patient weights in pediatric anesthesia can lead to significant medication excess and waste from single-use medication vials packaged for dosing in adults. Medication aliquots have been proposed as a strategy to decrease waste and therefore expense when using high-cost medications such as sugammadex. Appropriate dosing of sugammadex to antagonize neuromuscular block is based on the results of quantitative monitoring, though the use of these monitors is not routine. In this quality improvement project, we demonstrate cost savings from aliquoting sugammadex from large, single-use vials and using quantitative monitoring to guide accurate and appropriate dosing.

Methods

After institutional review and approval, patients receiving rocuronium neuromuscular block during their anesthetic care between October 10 and December 9, 2022, were included for analysis. Sugammadex aliquots were prepared under sterile conditions in the operating room pharmacy according to current compounding guidelines. Quantitative neuromuscular monitoring with electromyography-based monitors was used to guide accurate sugammadex dosing. Cost analysis included the actual savings achieved when aliquots were used instead of single-use vials, the potential savings if aliquots had been used as opposed to single-use vials, and the actual savings achieved when quantitative monitoring indicated that adequate spontaneous recovery was reached and sugammadex administration was not needed.

Results

A total of 200 patients were included in the analysis. In 73 patients, a 200 mg/2 ml vial of sugammadex was utilized, while 86 patients received sugammadex from pre-filled aliquot syringes of 50 mg/0.5 ml. Forty-one patients did not require sugammadex antagonism as they achieved spontaneous recovery to a train-of-four ratio ≥90%.

Conclusion

Administration of sugammadex from aliquots rather than manufacturer-packaged single-use vials, with dosing guided by quantitative neuromuscular monitoring, produced a net cost savings of approximately $46 per case and projected net annual cost savings of nearly $370,000 in our institution. Forty percent of the net cost savings came from confirmation by quantitative monitoring of adequate spontaneous recovery to a train-of-four ratio ≥90%.

## Introduction

Neuromuscular blocking agents (NMBAs) are commonly utilized in anesthesia care to facilitate tracheal intubation and optimize surgical exposure and surgical conditions. Recovery from these agents may occur spontaneously via drug metabolism and elimination, but more commonly it is facilitated by the administration of an antagonist such as neostigmine or sugammadex. The American Society of Anesthesiologists (ASA) recently published guidelines on the use, monitoring, and antagonism of the neuromuscular block that recommend sugammadex over neostigmine to antagonize deep, moderate, and shallow levels of block [1]. Unlike neostigmine, sugammadex can effectively and efficiently antagonize all depths of neuromuscular block and has in fact become the agent of choice for pediatric anesthesiologists who have trained since its introduction to the U.S. market in 2015 [2]. Its relatively high cost, however, has prevented its adoption for routine use in anesthesia care.

One strategy suggested to mitigate the expense of using high-cost medications is vial-splitting and repackaging [3]. This may be especially true for pediatric patients in whom dosing is weight-based and medications administered intraoperatively are commonly supplied in single-use manufacturer vials, packaged to account for appropriate dosing in the adult patient. The unused medication surplus in pediatric patients represents excess costs, especially when expensive medications are involved. Potential cost savings from aliquoting sugammadex have been reported, but this was done retrospectively without quantitative monitoring to properly guide the most appropriate dose and timing of this antagonist [4,5].

To understand the extent of excess sugammadex that is wasted and the potential for cost savings, we conducted a quality improvement project to aliquot sugammadex from large-dose vials into smaller pre-drawn syringes in a sterile pharmacy setting. We used quantitative electromyography monitors to determine appropriate sugammadex dosing [6] and to accurately assess the impact of vial splitting on cost savings in our institution. Portions of this article were presented previously as a meeting abstract at the International Anesthesia Research Society Annual Meeting on April 15, 2023. 

## Materials and methods

The project was conducted at Children's Hospital Colorado, Aurora, CO. Institutional review and approval were obtained for this quality improvement project (Children's Hospital Colorado Organizational Research Risk and Quality Improvement Review Panel, ORRQIRP #2208-3; informed consent was waived). This prospective project included all patients undergoing procedures with general anesthesia in the operating rooms and cardiac catheterization suites at a large pediatric tertiary care center between October 10 and December 9, 2022. Inclusion criteria were patients with planned rocuronium administration and subsequent sugammadex antagonism. Exclusion criteria were patients who were expected to remain intubated at the end of their procedure or undergoing after-hours or emergency procedures.

Sugammadex aliquots were prepared under sterile conditions in the operating room pharmacy according to USP <797> compounding guidelines [7] and stored in a centrally refrigerated location [8]. Aliquots were considered expired at nine days beyond the use date. For this project, a 500 mg/5 ml vial of sugammadex was divided into ten 50 mg/0.5 ml pre-filled syringes. The resulting medication cost was $21.01 per pre-filled syringe. Standard 200 mg/2 ml vials were also available for use and stocked in anesthesia workstations in each anesthetizing location per usual workflow, with a cost of $109.79/vial. 

Quantitative electromyography neuromuscular monitors (TetraGraph, Senzime™, Uppsala, Sweden) were utilized to determine appropriate sugammadex dosing based on the measured depth of block (Table 1) [1,9]. TetraGraph monitors were provided on loan for the purposes of this project, and Food and Drug Administration (FDA)-approved, single-use sensors were purchased at a cost of $27 each. Monitoring sites included adductor pollicis, abductor digiti minimi, or flexor hallucis brevis muscles. No facial muscle monitoring was performed as recommended by recently published guidelines [1].

**Table 1:** Depth of block and sugammadex dosing PTC: post-tetanic count; TOFC: train-of-four count; TOFR: train-of-four ratio

Depth of Block	Quantitative Monitoring Measurement	Sugammadex Dosing (mg/kg)
Complete	PTC = 0	No dosing recommendations
Deep	PTC ≥ 1 to TOFC 0	4
Moderate	TOFC 1 to 3	2-4
Shallow	TOFR < 0.4	2
Minimal	TOFR 0.4 to < 0.9	2
Adequate Recovery	TOFR ≥ 0.9	0

Data collected from each patient included age, weight, total rocuronium dose per case, depth of neuromuscular block prior to antagonism, the total amount of sugammadex administered, and the source of sugammadex (vial versus pre-filled syringe). Data was entered into an Excel spreadsheet and calculations were performed using Microsoft Excel (2021) (Microsoft Corporation, Redmond, Washington, United States), version 16.66.1. The use of pre-filled syringes, or aliquots, was encouraged but not mandated. Therefore, the total amount of sugammadex administered for each case was used to determine the corresponding number of aliquots or vials that would have been required. For instance, the number of aliquots used or needed for a case was determined by dividing the actual amount of sugammadex administered in milligrams by the amount of sugammadex in a single aliquot (50 mg/aliquot). The results of these calculations are rounded up to the nearest whole number to account for partial aliquots used. Likewise, the number of vials used or needed for a case was determined by dividing the actual amount of sugammadex administered in milligrams by the amount of sugammadex in a single-use vial (200 mg/vial), with the results rounded up to the nearest whole number to account for partial vials used.

Cost comparisons for each case were determined by calculating the actual cost of aliquots used versus the potential cost if vials had been used (gross savings) or the actual cost of vials used versus the potential savings if aliquots had been used (gross potential savings). If quantitative neuromuscular monitoring indicated that a train-of-four ratio (TOFR) ≥0.9 was achieved spontaneously by the end of the case, then an antagonist was not administered. For these cases, cost savings were calculated by comparing the actual sugammadex cost ($0) to the cost of 2 mg/kg sugammadex obtained from a single-use vial, considering current anesthesia practice where quantitative monitoring is not used routinely and pre-filled syringes are not available.

Considering that quantitative monitoring is not currently standard practice for most anesthesiologists, the potential expense of disposable TetraGraph sensors (TetraSens, Senzime) was also included in calculating the cost per case. The net savings for each case were determined by subtracting the cost of a single quantitative monitoring sensor ($27.00) from the calculated gross savings. Total gross and net savings for the project period were determined by summing the values obtained from individual cases. An estimated annual net savings to the institution was determined based on approximately 8,000 anesthetics/year in which rocuronium neuromuscular block and sugammadex antagonism are used.

## Results

During the project period, seven patients received neuromuscular blockade and sugammadex antagonism in the absence of quantitative monitoring. These patients were excluded from cost savings analysis as we were unable to verify appropriate sugammadex dosing as guided by quantitative neuromuscular monitoring. Two hundred patients were included in the analysis for this project. The mean age, weight, and medication dosing of this cohort according to sugammadex source is shown in Table 2.

**Table 2:** Demographics and dosing according to sugammadex source

	Sugammadex Source		No Reversal Required
	Aliquot	Vial	
Mean age (years)	8.6	15	12.7
Mean weight (kg)	31.8	60.8	51.4
Mean total rocuronium per case (mg/kg)	1.2	1.1	1.0
Mean total sugammadex per case (mg/kg)	2.5	2.9	0.0

A 200 mg/2 ml vial of sugammadex was used in 73 patients, while 86 patients received sugammadex from 50 mg/0.5 ml pre-filled syringes (aliquots). Forty-one patients did not require sugammadex antagonism as they achieved spontaneous recovery to a TOFR ≥0.9, assessed by quantitative neuromuscular monitoring at the end of their surgery (Table 3). 

**Table 3:** Sugammadex cost and savings analysis *: savings compared to using equivalent doses from vials for aliquot cases or dosing 2 mg/kg from a vial for cases not requiring reversal; **: vial savings represents the potential savings these cases would have obtained had the equivalent dose of sugammadex been sourced from aliquots; #: sugammadex drug cost savings minus the $27 per patient cost of a quantitative neuromuscular monitor single-use sensor.

	Sugammadex Source		No Reversal Required	Total
	Aliquot	Vial		
Cases	86	73	41	200
Cost of single unit	$21.01	$109.79	$0.00	--
Mean drug per case (mg/kg)	2.52	2.93	0.00	2.2
Mean drug cost per case	$1.66/kg	$2.52/kg	$0.00/kg	$1.63/kg
Gross savings	* $6,484.27	** $3,301.64	* $4830.76	$14,616.67
# Net savings	$4,162.27	$1,330.64	$3,723.76	$9,216.67
Gross savings per case:				$73.08
Net savings per case:				$46.08
Estimated Annual Net Savings (8,000 cases/year):				$368,666.80

A calculated net savings of nearly $5500 were seen when sugammadex was sourced from aliquots as opposed to vials (calculations involved the actual savings using aliquots used versus the potential cost if vials had been used, or the actual cost of vials used versus the potential savings if aliquots had been used). An additional net savings of $3723 were realized when quantitative monitoring identified patients who had achieved spontaneous recovery and did not require administration of sugammadex (Table 3). Over a year, with an estimated 8,000 anesthetic cases that require neuromuscular block, a net savings of $46 per case (accounting for the cost of disposable sensors) and an estimated annual net savings of nearly $370,000 were calculated. 

## Discussion

Appropriate dosing and re-dosing of NMBAs and their antagonists should be based on the results of quantitative monitoring, and recently published guidelines in adult patients recommend quantitative monitoring and confirmation of adequate recovery from neuromuscular block prior to tracheal extubation to prevent potential complications from residual weakness [1,9-11]. Unfortunately, quantitative neuromuscular monitoring has yet to become common practice, so administration doses of high-cost medications like sugammadex may be incorrect. The potential cost savings that could be realized with strategies such as vial splitting and repackaging of sugammadex therefore remain incompletely documented. For this quality improvement project, we utilized quantitative electromyography (EMG) monitors to accurately assess the depth of the neuromuscular block and determine the need for antagonism and its proper dosing based on the depth of the block at the time of administration. We found potential cost savings of almost $370,000 per year if using sugammadex aliquoted from large, single-use vials into smaller pre-filled syringes if the medication timing and dosing administration were guided by quantitative monitoring.

In pediatric anesthesia, sugammadex has become the preferred agent to antagonize amino-steroidal neuromuscular blocking agents [2], especially by anesthesiologists who recently completed their training. Within our institution, sugammadex use is unrestricted and is the agent of choice when antagonizing steroidal neuromuscular block. An estimated 8,000 patients per year currently receive sugammadex, and a review of data between 2017 and 2019 revealed that 80% of these patients received a total dose of 50 mg or less. The smallest single-use vial currently available from the manufacturer is 200 mg/2 ml, resulting in enormous wastage in an era of supply chain issues and drug shortages. Mpody et al. recently discussed the economic implications of sugammadex wastage when single-use, 200 mg/ml vials are used in pediatric anesthesia practice, noting a potential $14,000,000 worth of excess drug that may have been discarded during their five-year study period [4]. They proposed cost mitigation strategies, including regulations on manufacturers to produce dosing packages appropriate for pediatric use or agreements in which the cost of unused (and wasted) drugs due to current packaging restraints would be refunded. These strategies would unfortunately require regulatory mandates or voluntary changes in pharmaceutical industry practices, which have yet to be realized and over which institutions and practitioners have little control. 

Amaya et al. performed a retrospective study of potential cost-savings by splitting 200 mg sugammadex vials into 50 mg and 100 mg aliquots with a corresponding cost of $106, $53, and $26.50 per 200 mg, 100 mg, and 50 mg doses, respectively. A third of patients in their analysis received ≤50 mg, and they found the average cost of sugammadex per patient was $113 when using 200 mg vials, $81 with 100 mg aliquots, and $68 when 50 mg aliquots were used [5]. However, the use of quantitative monitoring and the rationale for their sugammadex dosing were not reported. In the absence of quantitative monitoring, sugammadex may have been overdosed or underdosed in any given case. This imprecise dosing regimen may impact cost estimates if patients who are fully recovered are still receiving antagonists, or more importantly, from a patient safety perspective, if those patients who are not fully recovered are assumed to be and are not given an antagonist. 

Dosing indications for sugammadex are based on quantitative monitoring [9]. Indeed, quantitative assessment is the only way to determine if patients are adequately recovered and therefore do not need to receive an antagonist to facilitate recovery. Common (and inadequate) practice, however, is to assess patients by clinical signs or subjectively with a peripheral nerve stimulator. These qualitative assessments require a subjective judgment of a patient’s muscular strength, often leading to an overestimation of recovery as the human senses are unable to distinguish shallow levels of the residual block from complete recovery [12]. Sugammadex may therefore be omitted when it is indicated, resulting in residual neuromuscular weakness and its potential complications [13,14]. Alternatively, full recovery may be achieved spontaneously and administration of sugammadex at this level of recovery can lead to exposure to unnecessary medications with potential health risks [15] and increased cost. In utilizing quantitative monitoring for this project, we were able to accurately assess neuromuscular block in our patients and appropriately administer sugammadex at doses of either 2 mg/kg or 4 mg/kg according to the measured depth of block. Additionally, we found that 41 patients did not require antagonism with sugammadex at the end of their surgery as they had spontaneously achieved a TOFR ≥0.9. This group, while comprising 20% of the project cohort, accounted for 40% of the calculated net savings (Table 3). Estimates of these potential savings cannot be made in the absence of quantitative monitoring and therefore were not available in prior studies [4,5]. Interestingly, we did not define how neuromuscular block was administered or maintained during this project period. It is therefore possible that using quantitative monitoring not only to determine appropriate sugammadex dosing at the end of a case, but to achieve and maintain adequate neuromuscular block during a case, might increase the proportion of patients requiring smaller doses of sugammadex, or even no sugammadex administration.

The recently published ASA practice guidelines recommend the use of quantitative neuromuscular monitors when neuromuscular blocking drugs are used [1]. For this project, we chose EMG-based monitors. EMG monitors measure compound muscle action potentials as opposed to acceleromyography (AMG)-based monitors that measure muscle acceleration, usually at the thumb. AMG requires unimpeded motion of the monitored muscle (thumb), which is not typically feasible after surgical positioning of children in which the arms are commonly tucked to their sides under surgical drapes [16,17]. EMG is therefore ideally suited for neuromuscular monitoring in children, but it does require a specialized disposable sensor (TetraSens), which is a recurring cost of $27.00 in this project. If AMG monitoring were feasible in practices that allow unencumbered access to the patient’s hand, the use of standard ECG electrodes (approx. $0.2-0.3 each) would mitigate this cost. Despite the inclusion of disposable sensor costs in our analysis, though, we found that sourcing sugammadex from aliquots compared to single-use vials still produced a net savings of $46 per case when the administration of a reversal agent was guided by quantitative monitoring (Table 3).

Aside from the potential for recurring charges with sensors, we recognize the acquisition cost of quantitative monitors may, for some institutions, be prohibitive. For the purposes of this project, monitors were provided on loan and so were not an expense factored into our analysis. However, the projected savings for our institution with sugammadex dosed from aliquots and guided by quantitative monitoring are still substantial. For our institution, an estimated 8,000 cases per year receiving neuromuscular block that is antagonized with sugammadex could amount to nearly $370,000 annually. The savings more than offset the one-time cost of monitor acquisition. For instance, if the conservative cost for quantitative monitoring were $2,000 per unit, our estimated net annual savings could provide quantitative monitors for 185 anesthetizing locations, far in excess of the number of our 42 anesthetizing locations.

We acknowledge the limitations of this QI project. First, cost savings may not be universally applicable due to institutional differences with respect to pharmacy staffing, workflow, and cost centers. Our pharmacy included aliquoting into their existing workflow, so no additional “cost” to aliquoting was involved. Amaya et al. estimated the cost of vial splitting to equate to approximately 0.1 full-time equivalent (FTE) of pharmacist time [5]. For this project, our operating room pharmacist independently reported their time invested in this activity was similar. The compounding, labeling, and restocking of sugammadex aliquots were estimated to require two to four hours per month. While vial-splitting may not have a significant impact on pharmaceutical budgets for large hospitals or health systems [18], we acknowledge the barriers that may be present in some facilities.

The use of quantitative neuromuscular monitoring to guide appropriate assessment and dosing of sugammadex was also an integral part of maximizing cost savings and supporting patient safety. During this project, we circulated through the operating rooms each day to ensure the use of monitoring in cases where rocuronium was used. However, the introduction of quantitative monitors and maintaining their use in routine practice is extremely difficult [19,20]. Indeed, despite our daily presence and reminders during the project period, quantitative monitors were not documented as being utilized in 3.5% of cases. While this number may be quite low, considering that only 67% of pediatric anesthesiologists report routine use of monitoring since the introduction of sugammadex in the US [2], it underscores the challenges of adopting quantitative monitoring into routine practice. Weigel et al. [20] included education, reminders, performance feedback, and the metric of documented monitoring in their credentialing process as strategies to support practice change. Integrating quantitative monitors into the electronic medical record for reliable documentation and utilizing dashboards for individual provider feedback may also be strategies to support adoption. If not used routinely, potential cost savings may be reduced. Additionally, patient safety could be impacted by the omission of indicated antagonism [13] or by unnecessary exposure to medications and their potential for hypersensitivity/anaphylaxis, bradycardia, and asystole [21].

Finally, our results focus on mitigation of the high cost of sugammadex, employing vial-splitting and proper dosing guided by quantitative monitoring, but do not address cost savings that might be realized if we used the traditional cholinesterase inhibitor, neostigmine, for antagonism. Medication acquisition costs for sugammadex have been shown to be 10-20 times higher than for neostigmine [22]. The recently published ASA guidelines on the use, monitoring, and antagonism of neuromuscular block recognize neostigmine as a reasonable alternative to sugammadex for antagonizing minimal block [1], and a protocol for its successful use has been described [23]. Within our institution, sugammadex is unrestricted and is the antagonist of choice for all levels of block. However, we appreciate that the use of neostigmine could offer further cost-savings beyond those attained with vial-splitting and re-packaging of sugammadex described by this project, as long as neostigmine administration was guided by quantitative monitoring. Our estimated pharmacy costs for neostigmine range from $3.77- $8.97 for a single-use 10 mg vial and $1.65-$2.33 for a single-use 2 mg vial of glycopyrrolate, depending on the vendor source. This would equate to a cost range of $5.42 - $11.33 if dosing required a single-vial each of neostigmine and glycopyrrolate. Compared to a cost of $21 for a pre-filled syringe of neostigmine/glycopyrrolate mixture or a $109 single-use 200 mg vial of sugammadex, antagonism of minimal block with neostigmine and glycopyrrolate might therefore be accomplished at one-fourth to one-twentieth of the cost. Approximately one-fourth of our cohort (52 patients) were noted to be at a minimal level of a block at the time of sugammadex administration, representing the additional potential for savings, though the cost comparisons for neostigmine versus sugammadex when considering patients who achieve adequate spontaneous recovery would lower these projections. It should be noted that the appropriate assessment of neuromuscular block for dosing neostigmine and ensuring adequate recovery following its administration requires quantitative monitoring, so this component of our cost-savings analysis would not change. Furthermore, the cost comparisons for neostigmine versus sugammadex when considering factors such as complications of residual or recurrent neuromuscular block or efficient operating room and recovery room exit times are complex and beyond the scope of this project. Future studies incorporating the appropriate use of sugammadex (sourced from vial-splitting and re-packaging) or neostigmine guided by quantitative monitoring of neuromuscular block are encouraged.

## Conclusions

This quality improvement project demonstrated that an estimated annual cost savings of nearly $370,000 might be realized through aliquoting sugammadex from 500 mg/5 ml vials into 50 mg/0.5 ml pre-filled syringes, as compared to drug administered from 200 mg/2 ml vials. These savings were based on the results of quantitative neuromuscular monitoring, which is necessary to determine the depth of neuromuscular block and guide appropriate sugammadex dosing. We stress that quantitative monitoring is the only way to determine if sufficient spontaneous recovery from neuromuscular block has occurred and sugammadex can be omitted, which accounted for 40% of the net savings reported in this project.
